# Medical Impertinence

**Published:** 1895-04

**Authors:** 


MEDICAL IMPERTINENCE.—Several of our exchanges have noticed with comment a resolution adopted by the New York County Medical Association, December 17th, protesting against the appointment by Governor-elect Morton of a homœopathic practitioner to supersede Dr. Joseph D. Bryant in the position of Surgeon-General of the State.

We in New York are so familiar with the impertinence of this society, that we no more expect the professional decencies of gentlemen from its members than we do any other music but that of a bray from a jackass. It may be a comfort to our exchanges to know that this association is a little party of bigots, small in number and still smaller in influence and talent, who cut loose from the main body in 1892, when the split occurred on the code question, and still adhere to the old iron-bound code, while the great body of the school, represented by the New York County Medical Society, claim to be governed by no code but that of the gentleman. It was the New York County Medical Association, and not the New York County Medical Society, which was guilty of this impertinence.—*New York Medical Times.*

